# Dengue outbreaks in the COVID-19 era: Alarm raised for Asia

**DOI:** 10.1371/journal.pntd.0009778

**Published:** 2021-10-08

**Authors:** Xinting Lu, Hilary Bambrick, Puntani Pongsumpun, Pandji Wibawa Dhewantara, Do Thi Thanh Toan, Wenbiao Hu

**Affiliations:** 1 School of Public Health and Social Work, Queensland University of Technology, Brisbane, Australia; 2 Department of Mathematics, Faculty of Science, King Mongkut’s Institute of Technology Ladkrabang, Bangkok, Thailand; 3 Center for Research and Development of Public Health Effort, National Institute of Health Research and Development, Ministry of Health of Indonesia, Jakarta, Indonesia; 4 School of Preventive Medicine and Public Health, Hanoi Medical University, Hanoi, Vietnam; Minia University, EGYPT

Havoc associated with the novel Coronavirus Disease 2019 (COVID-19) pandemic is placing unprecedented pressure on healthcare and public health system worldwide. With medical resources and personnel being directed to battle COVID-19 globally, the pandemic may have grave repercussions in the resurgence of other infectious diseases such as dengue fever, especially in resource-limited countries with both endemic dengue and intense COVID-19 transmission.

During the COVID-19 pandemic, dengue cases spiked in Brazil, introducing an added burden on already fragile healthcare systems [1]. The COVID-19 pandemic coincided with a resurgence of dengue in Brazil, raising concern for countries in Asia where dengue occurring across tropical and subtropical regions of Asia accounts for approximately 70% of the global disease burden [2]. Considering the region’s extremely high burden of dengue, its climate change vulnerability and—imminently—the beginning of the monsoon season, there needs to be concerted actions to prevent large dengue outbreaks during and beyond the COVID-19 pandemic.

Preventing or reducing dengue virus transmission depends primarily on mosquito vector controls including interrupting human–vector contact. At a time of continuous lockdowns, when public health staff are diverted to control COVID-19 transmission and community engagement focused on the pandemic, routine mosquito vector surveillance and control programs are discontinued or paused in many countries [3], which will impair dengue control and prevention.

A study of India found that the immature density of *Aedes* mosquito drastically increased during the COVID-19 lockdown due to paused vector control programs [4], and an increased density of vectors was also reported in Malaysia during the COVID-19 lockdown, which has led to increased dengue incidence [5]. During the lockdowns, when human movement is limited to and around own homes, perversely, human–vector contact may be enhanced, resulting in an increased risk of exposure and virus transmission. This impact is likely to be even more pronounced in settings where dengue virus transmissions primarily occur in or between households, rather than an occupational setting. A study quantified the impact of lockdowns on dengue incidence and suggested that a rise in dengue cases associated with lockdowns in Thailand; however, no significant impact on dengue transmission was found in Singapore and Malaysia [6]. A robust study in Thailand found that 60% of dengue cases living less than 200m apart came from the same transmission chain, providing strong evidence that residences play a primary role in dengue virus transmission [7]. Conversely, in areas where mosquito density is high in public spaces, like workplaces or schools, decreased dengue transmission may occur during a lockdown, such as occurred among migrant workers in Singapore [8].

The rapid spread of dengue viruses from endemic to nonendemic countries is associated with international airline travel [9]. Stringent international air travel bans during the COVID-19 pandemic implemented globally have resulted in sharp decreases in dengue cases in countries where dengue is almost predominantly imported, such as Australia, due to a precipitous decline in the importation of exotic pathogens by infected overseas travelers [10]. However, within dengue endemic countries, international air travel bans are unlikely to alter local dengue transmission patterns substantially; nationally, human movements along busiest national road of the country have been found to play a major role in the spread of dengue infection in dengue endemic countries [11]. Impacts of movement restrictions induced by lockdown measures on dengue transmission plausibly vary between countries, with differing dengue transmission dynamics and severities.

Most dengue endemic countries are low- and middle-income countries. Lower socioeconomic conditions with large numbers of people living in densely populated areas in poorly constructed housing without adequate clean water and sanitation tend to promote *Aedes aegypti* populations and lead to dengue outbreaks [12]. As the COVID-19 pandemic continues to unfold, poorer countries and lower socioeconomic groups are now disproportionately affected [13], with already disadvantaged conditions exacerbating the potential for dengue epidemics and overlapping with other infectious disease outbreaks. This is occurring alongside the mounting challenges of climate change, when intensifying and increasingly frequent extreme weather events are coinciding with the disruption caused by the COVID-19 to systems and services, including, but not limited to, those directly in the health sector, with potentially multiplicative outcomes for population health [14].

Lockdown measures implemented for the COVID-19 pandemic may also have interrupted already incomplete dengue case detection and reporting, especially in poorer endemic countries that lack effective dengue surveillance systems. Passive mechanisms of dengue surveillance and case detection largely depend on dengue-infected individuals presenting to the healthcare system and the provider subsequently reporting the case to authorities. When combined with insufficient access to dengue diagnostic tests, underestimation of cases is likely. Under lockdown measures, people with dengue symptoms, especially if mild, may delay or avoid seeking healthcare [15], while dengue diagnostic testing competes with prioritized testing for the coronavirus. Further, it is possible that prior to blood test confirmation, misdiagnosis between dengue fever and COVID-19 could occur because they share similar clinical manifestations in the early stages [3,16]. Conversely, because of the need to distinguish febrile illnesses in the pandemic, there may be an increase in testing for dengue, which, in turn, could uncover more dengue cases. A potential complicating factor however is of lower sensitivity and specificity of the dengue rapid tests due to possible cross-reactivity between the coronavirus and dengue virus antibodies [17]. In Brazil, observed increase in dengue cases reported during the COVID-19 pandemic may be partially the result of extended testing compared with years before the pandemic [1]. However, this hypothesis is needed to be confirmed by dengue testing data.

**Fig 1:**
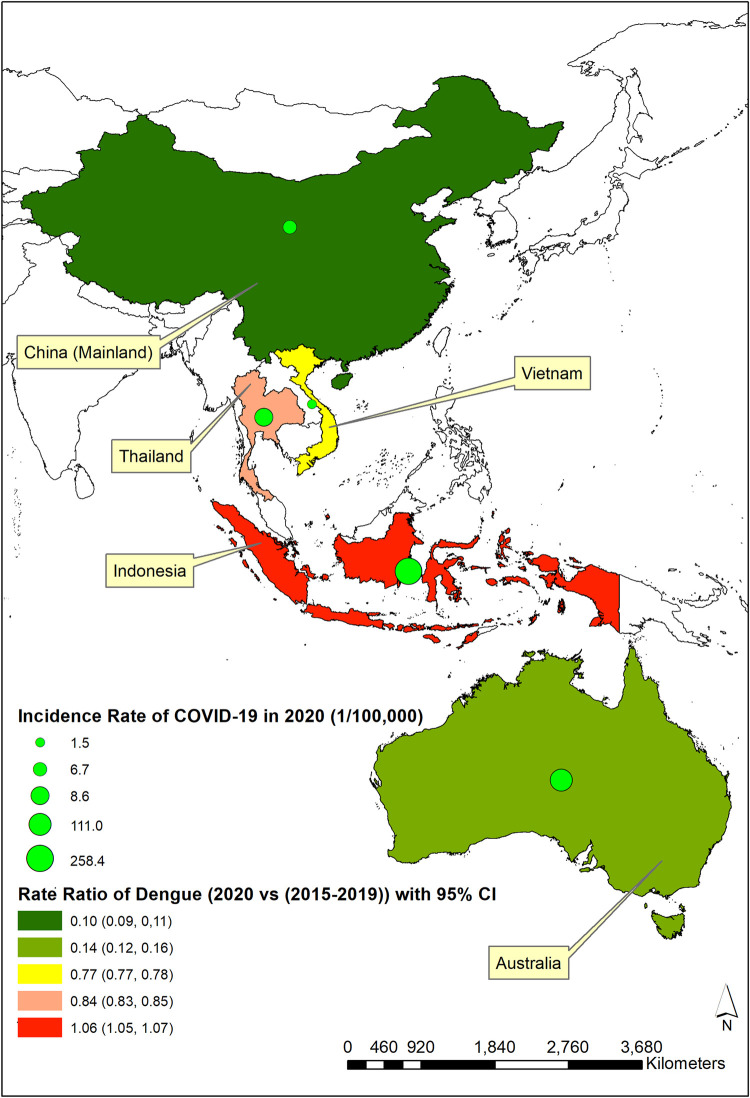
Dengue IRR (95% CI) (2020 versus 2015–2019 average) in the context of incidence of COVID-19 (per 100,000 population) in 2020 for Australia, China (Mainland), Indonesia, Thailand, and Vietnam. ArcGIS software (version: 10.8) was used to create the map. All digital boundary shapefiles are in the public domain (Thailand: https://data.humdata.org/dataset/thailand-administrative-boundaries; Vietnam: https://data.humdata.org/dataset/viet-nam-administrative-boundaries-polygon-polyline; Indonesia: https://data.humdata.org/dataset/indonesia-administrative-boundary-polygons-lines-and-places-levels-0-4b; China (Mainland): https://data.humdata.org/dataset/china-administrative-boundaries; and Australia: https://www.abs.gov.au/statistics/standards/australian-statistical-geography-standard-asgs-edition-3/jul2021-jun2026/access-and-downloads/digital-boundary-files). CI, confidence interval; COVID-19, Coronavirus Disease 2019; IRR, incidence rate ratio.

**Fig 2:**
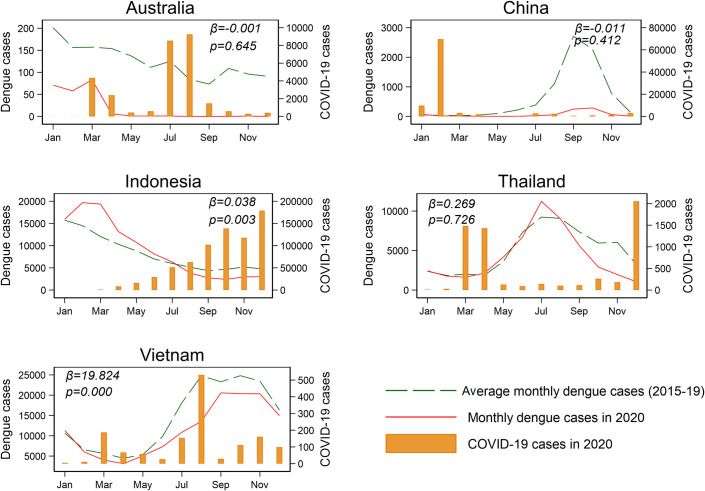
Reported monthly case numbers of dengue in Australia, China (Mainland), Indonesia, Thailand, and Vietnam for 2015–2019 average, and in 2020, shown against monthly cases of COVID-19 in 2020. COVID-19, Coronavirus Disease 2019.

To explore potential impacts of the COVID-19 pandemic on dengue, we analyzed reported case numbers for dengue during 2015 to 2020 and for COVID-19 in 2020 from 5 Asia-Pacific countries: Australia, China (Mainland), Indonesia, Thailand, and Vietnam. Dengue incidence rate ratio (IRR) (i.e., annual incidence of dengue in 2020 divided by the average incidence of dengue during 2015 to 2019) was calculated to represent and compare the extent of changing incidence of dengue between 2020 and the previous 5 years across the study countries in the context of the COVID-19 pandemic. The incidence rate for COVID-19 in 2020 was calculated to demonstrate the severity of pandemic in different countries (Fig 1). Fig 1 showed that IRR substantial decreased in China (IRR: 0.10, 95% confidence interval (CI): 0.09 to 0.11) and Australia (IRR: 0.14, 95% CI: 0.12 to 0.16), slightly decreased in Vietnam (IRR: 0.77, 95% CI: 0.77 to 0.78) and Thailand (IRR: 0.84, 95% CI: 0.83 to 0.85), and slightly increased in Indonesia (IRR: 1.06. 95% CI: 1.05 to 1.07). To further test the impact of COVID-19 on dengue transmission, we fitted a regression model between monthly COVID-19 cases and monthly dengue cases after adjusting for seasonality (i.e., monthly number of dengue cases in 2020 at *i* month − average monthly number of dengue cases during 2015 to 2019 at *i* month ~ number of COVID-19 cases at *i* month). The results suggested that COVID-19 was significantly and positively associated with dengue in Indonesia (*β* = 0.038, *p* = 0.003) and Vietnam (*β* = 19.824, *p* = 0.000) (Fig 2). No statistically significant negative association was found in any of these countries.

In Australia, dengue outbreaks are usually triggered by imported cases from endemic countries, with a subsequent seasonal outbreak that includes local transmission [18]. Public health control measures implemented in the early months of the COVID-19 pandemic, especially the country’s stringent international air travel ban, resulted in a sharp decrease in dengue cases nationally [10]. A recent research in China suggested that dengue remains as an imported disease and is not endemic in Guangzhou [19]. However, in countries like Indonesia, Thailand, and Vietnam where dengue fever is predominantly locally acquired and transmitted, and the spread of infection is primarily determined by local vector mosquito density driven by local suitable weather conditions and seasonality, lockdown measures and any international air travel restrictions and quarantine are unlikely to alter local dengue transmission patterns substantially. Moreover, it is interesting that the significant positive relationship between monthly dengue cases and COVID-19 cases were found in Vietnam and Indonesia. This finding might indicate the potential impacts of the pandemic in these two countries, howbeit more rigorous studies are required to confirm our research.

Although this observational analysis may be vulnerable to confounding due to a variety of transmission dynamics and surveillance capacity in different countries, these data support that the pandemic may have impacts on dengue transmission, with different effects across countries. A research conducted in Singapore found an increased dengue transmission during the COVID-19 pandemic [20], while others conducted found decreased transmission [19,21].

As the world remains focused on managing the global COVID-19 pandemic, we need to remain mindful that the sustained efforts are needed to prevent the resurgence of other infectious diseases. This is of concern for dengue endemic countries in the Asia-Pacific region, considering the monsoon season in the Indian subcontinent and surrounding regions and the serious situation of the COVID-19 pandemic in this region. Based on recent research and our observation in Asia-Pacific region, some very specific points should be taken care of as follows: (1) continuing to monitor and assess the possibility of reimportation and resurgence of dengue where dengue is not endemic, especially during outbreaks of novel serotypes in neighboring countries [18]; (2) paying close attention to the case number in tropical and subtropical countries when heatwaves occur, as these may raise the risk of dengue outbreaks [22], and weather forecasting to inform timely public health prevention; (3) ensuring effective dengue disease surveillance and enhanced diagnostic testing capacity and allocation of resources for clinic health personnel training on distinguishing dengue from COVID-19 in the context of coepidemics [23]; this is especially challenging in low- and middle-income countries where highly sensitive diagnostic tests are not available; and (4) calling for more researches for a better understanding of different impacts of the COVID-19 pandemic on dengue transmission and develop an integrated early warning system.
